# Seeing right through it: X-ray analyses of *Uniola paniculata* L. spikelets reveal seed production patterns across a wide spatial distribution

**DOI:** 10.1093/aobpla/plaf021

**Published:** 2025-04-12

**Authors:** Héctor E Pérez, Tia Tyler, Michael E Kane

**Affiliations:** Environmental Horticulture Department, University of Florida, 2047 IFAS Research Drive, Gainesville, FL 32611, United States; Environmental Horticulture Department, University of Florida, 2043 IFAS Research Drive, Gainesville, FL 32611, United States; Environmental Horticulture Department, University of Florida, 2043 IFAS Research Drive, Gainesville, FL 32611, United States

**Keywords:** abundant center model, barrier islands, coastal dunes, eco-spatial gradient, latitudinal gradient, phylo-geographic gradient, panicle density, seed quality

## Abstract

Coastal dunes represent globally important ecosystems heavily impacted by human activities and requiring nature-based restoration solutions. Plants with dune building and stabilizing traits typically represent the dominant vegetation. Such species can have ranges extending >1000 km albeit in fragmented populations. Seeds of dominant species are in high demand for establishing dune restoration planting material, but supply may be limited given variable *in planta* seed production across their range. The broad geographic occurrence of such species presents opportunities to question the influence of various abiotic and biotic gradients on seed production while providing answers that can inform seed-based restoration efforts. We modelled seed production of *Uniola paniculata* over a 2-year period from 17 populations spread over 12° of latitude (ca. 1357 km). Panicle density and dune type were strong predictors of normal seed production and number of seeds per spikelet. Interactions between eco-spatial zonation or haplotype and collection year were evident regarding the number of seeds per spikelets, but the effects were mostly negligible. Likewise, latitude and drought intensity yielded small-to-medium effects on the number of seeds per spikelet. The proportion of abnormal seeds was not unusual for wild species, and panicle density was not a strong predictor of this response. We hypothesize that a threshold panicle density exists below which seed production decreases substantially. Practitioners should assess relative panicle density at donor sites when creating seed collection plans and may consider sites with low panicle density as priority augmentation targets.

## Teaser

Many geographical, environmental, and biological factors influence a plant's ability to produce seeds. We found that the relative amount of sea oats (*Uniola paniculata* ) flowering structures has the largest effects on seed production of this crucial coastal dune-building species. Such information can be beneficial for dune restoration programs. Future studies aim to more fully quantify the flowering structure to seed production relationship.

## Introduction

Sandy coastal dune systems are comparatively minor in terms of global areal extent, yet these highly dynamic landforms play major roles in various ecological and geomorphological processes, while providing benefits to myriad species including humans (Stutz and Pilkey 2011; Zhang and Leatherman 2011; Martínez *et al.* 2013; Pérez-Maqueo *et al.* 2013; Boynton *et al.* 2020). Dune system morphology is typically described by zones extending landward from the ocean-fronting beach (Doing 1985; Johnson and Barbour 1990; Knight *et al.* 2011). The foredune is the first dune encountered when moving landward past the wet and dry beaches. Upright, perennial grasses with dune-building traits typically dominate the foredune. The foredune can then be followed by a series of relict dunes forming a relatively stable rear-dune complex of ridges and swales. Pioneer grasses give way to forbs, other low-growing grasses, and sporadic, small, woody, or non-woody shrubs in the rear dune (Wagner 1964; Johnson and Barbour 1990; Knight *et al.* 2011).

Coastal dunes in the USA primarily occur on barrier islands that extend about 3700 km while occupying about 6800 km^2^ along the Gulf and Atlantic coasts (Zhang and Leatherman 2011). The extent of these islands and associated dune systems creates an opportunity to address questions related to plant trait variation across a broad geographical gradient (De Frenne *et al.* 2013). However, as of 2000, nearly 1.4 million people inhabited barrier islands in the USA (Zhang and Leatherman 2011). This certainly represents an underestimation as states along these coasts experienced strong population growth since 2000 (USCB 2023). Increased levels of human activity have led to vast dune system alteration and fragmentation with associated decreases in natural vegetation abundance (Johnson and Barbour 1990). Subsequently, natural erosion and accretion processes are interrupted. For example, nearly all barrier islands in Florida are classified as critically eroded (Clark and Weeks 2023). The high degree of erosion along these coasts, coupled with previous or impending damage from strong storms and projected sea-level rise, culminates in a crucial need to rebuild dune systems. This is often accomplished via a combination of beach re-nourishment and dune restoration activities. Restoration plans frequently require establishment of natural vegetation to enhance dune building and stabilization processes (Martínez *et al.* 2013; Pérez-Maqueo *et al.* 2013).

**Figure 1.**
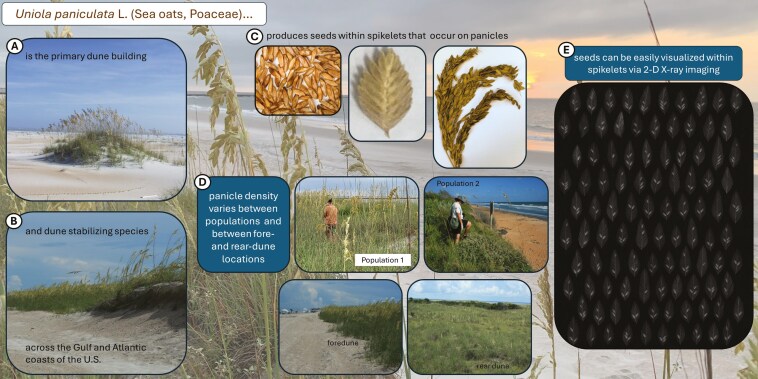
Visual representation of *Uniola paniculata* ecological significance (A, B); From left to right: seeds, spikelets, and panicles (C); variation of panicle density (D); and spikelet X-ray image example (E).

*Uniola paniculata* L. (Poaceae) is the primary dune building and stabilizing plant in the southeastern USA and the dominant species on foredunes (Fig. 1A and B). It produces mature seeds within spikelets by late September to early October throughout its range (Fig. 1C and D). Although *U. paniculata* is rhizomatous, unassisted establishment in the dunes only occurs via regeneration from seeds. However, *U. paniculata* is reportedly a poor seed producer (Wagner 1964; Hester and Mendelssohn 1987; Lonard *et al.* 2011). Nonetheless, seeds of this species are in high demand to establish greenhouse-grown seedlings for dune restoration projects. Yet, variable seed production throughout the *U*. *paniculata* range can hamper seed collection efforts and ultimately limit plant production as part of seed-based restoration programs. This is important as policy mandates require, or guidelines recommend, sourcing seeds from the same or nearby populations where re-vegetation is to occur (Hufford and Mazer 2003; McKay *et al*. 2005; Broadhurst *et al*. 2008; Breed *et al*. 2013).

Moreover, many native species used in restoration practice are known to produce copious empty seeds. For example, Pérez (2014) found that *Aristida stricta* Michx. [Poaceae] produced 55% to 90% empty seeds. Similarly, Kärkkäinen (1999) demonstrated that mean seed abortion varied between about 27% and 32% for naturally pollinated and experimentally crossed *Pinus sylvestris* L. cones. The presence of such abnormal seeds detracts from seed-based restoration efforts by decreasing seed lot quality. Practitioners will then have to consider adding overcollection margins to compensate for proportions of empty seeds, conditioning lots to remove abnormal seeds, and calculating seeding rates with larger overage factors or re-seeding during production to propagate sufficient plant materials for re-vegetation efforts. All these actions increase costs throughout the restoration process.

Therefore, we set out to clarify variation in *U. paniculata* seed production across its southeastern distribution. To accomplish this, we collected mature spikelets over a 12° latitudinal gradient and then visualized seeds within spikelets using 2-D X-ray radiography. A gradient of this length should be sufficient to capture potential differentiation among sites that may contribute to variation in seed production phenotypes (Hester and Mendelssohn 1987; Sagarin and Gaines 2002; Jump and Woodward 2003; Ayari *et al.* 2011; De Frenne *et al.* 2013; Daco *et al.* 2021). However, latitude by itself can be a poor predictor for characterizing trait variations given that other environmental factors may also vary across a wide spatial distribution (De Frenne *et al.* 2013). Therefore, we included eco-spatial, environmental, and phylo-geographic covariates in our analyses. We asked two main questions. First, how does the production of normal seeds, number of seeds per spikelet, and proportion of normal to abnormal seeds vary across the *U*. *paniculata* range within the USA? Second, which covariate(s) account for such variation. Information of this type could be beneficial for a clearer understanding of factors that drive seed production for widely distributed species from coastal dune systems and enhancing seed-based restoration programs.

## Materials and methods

### Spikelet collection and post-harvest handling

**Figure 2.**
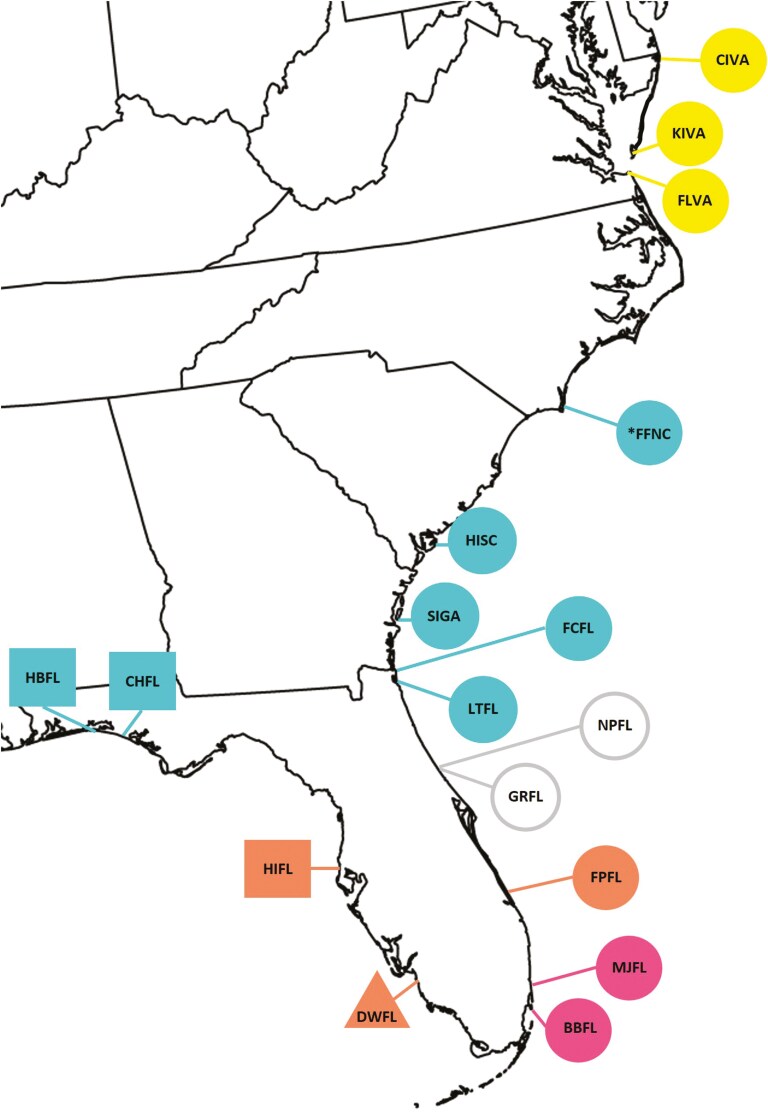
*Uniola paniculata* collection locations along the Gulf and Atlantic coasts of the United States. Circles, squares, triangle, and represent haplotypes A, C, and D, respectively, as defined by Hodel and Gonzalez (2013). Symbol colours denote Generalized Provisional Seed Zones 1 = yellow, 2 = blue, 3 = grey, 4 = orange, and 5 = pink. Open and filled symbols show populations with low and high panicle density, respectively.

Our collections occurred from 24 September through 5 October 2018 and 15 September through 4 October 2019. We used pruning shears to harvest one to three panicles per plant from a minimum of 30 widely spaced (>1 m) plants growing on the foredune at each site. We harvested panicles from the same 16 sites in both years (Fig. 2; Supplementary Table S1). We also collected panicles in rear dune locations from three populations in 2018 and five populations in 2019 (Supplementary Table S1). We allowed panicles to air dry for 3 days inside a non-climate-controlled warehouse, then hand-stripped spikelets into paper bags and transferred these to the laboratory. We thoroughly mixed spikelets within each bag and then randomly selected 100 spikelets per site and collection year combination (effect size = 0.15, *α* = 0.05, 1−*β *= 0.99; *df* = 31; Faul *et al.* 2007).

### Spikelet X-ray imaging

We arranged the spikelets from each site on laminating sheets (73601, Avery Products Corp., Brea, CA, USA) and sent these to the US Forest Service National Seed Laboratory for X-ray imaging. The machine (Ultrafocus, Faxitron Inc., Tucson, AZ, USA) was set to 1× magnification, 0.3–0.4 mA, 25–32 kV, and 5–8 s exposure time. We examined all digital radiographs (Fig. 1E) using photo viewing software (Photos, Microsoft Corp., Redmond, WA, USA).

### Data collection and analyses

We conducted statistical analyses using SAS^®^ software (v. 9.4, SAS Institute Inc., Cary, NC, USA).

#### Eco-spatial, environmental, and phylo-geographic covariates

We gathered eco-spatial, environmental, and phylo-geographic covariates in addition to the latitude of site occurrences (Supplementary Fig. S1). We grouped populations by general provisional seed zones (GPSZ; Fig. 2) proposed by Bower *et al.* (2014). The GPSZ divides the contiguous United States by established eco-regions, and climatological data, explains a considerable amount of variation in traits related to plant fitness, and serves as a basis for seed transfer zones that minimize the risk of maladaptation (Bower *et al.* 2014).

We then created two 5-point drought indices (DI) with data reported by the US Drought Monitor (NDMC 2024) during the vegetative to flower (VFDI) and flower to seed production (FSDI) pheno-phases for each *U. paniculata* collection site (Supplementary Table S1). The vegetative to flowering (i.e. March to May) and flower to seed (i.e. May to early October) phases encompass re-sprouting in the spring to floral initiation and post-floral development followed by anthesis through seed maturation, respectively (Wagner 1964; Harper and Seneca 1974; Colosi 1979; Hester and Mendelssohn 1987; Lonard *et al.* 2011). These phases lasted about 4–12 and 20–22 weeks, respectively, depending on location and aligned with previously cited observations. We used the formula $\frac{\sum \left(X\cdot D_{5}+X\cdot D_{4}+X\cdot D_{3}+X\cdot D_{2}+X\cdot D_{1}\right)}{W_{tot}}$ to calculate the drought indices where $W_{tot}$ = the total number of weeks for a selected pheno-phase of a given location; *X* = the number of weeks during the pheno-phase that a population was exposed to a specific level of drought; and levels of drought are denoted by $D_{5}$ = exceptional drought, $D_{4}$ = extreme drought, $D_{3}=severe$ drought, $D_{2}$ = moderate drought, $D_{1}$ = 1 abnormally dry conditions. We also grouped populations by haplotypes (Fig. 2) according to analyses reported by Hodel and Gonzales (2013). These authors created chloroplast-DNA-based haplotype network maps to investigate the phylo-geographic history of *U. paniculata* across the southeastern USA.

Preliminary data analysis indicated strong correlations between several covariates (Supplementary Fig. S1). Therefore, we opted to develop two primary models. The first model excluded all eco-spatial and environmental variables that correlated strongly (Pearon’s *r* ≥ 0.50) with the GPSZ variable. The second model substituted latitude for GPSZ. Both models included haplotype (Fig. 2), VFDI, FSDI, and collection year given the weak correlation of these variables to GPSZ or latitude. We also accounted for all two-way interactions with the collection year. We utilized a dataset that grouped spikelets according to collection from fore- or rear-dune systems (Fig. 1D; Supplementary Table S1) in a separate analysis. The resultant model consisted of dune, collection year, and dune × year interaction.

#### Total normal seed count per 100 spikelets

We counted the total number of fully developed, normal seeds (Supplementary Fig. S2), produced within spikelets and then assessed counts by fitting a generalized linear model with a Poisson distribution and log link function. However, overdispersion was evident. Therefore, we employed a negative binomial distribution (Hilbe 2011). The degree of overdispersion was greatly reduced, but it remained problematic (e.g. Pearson *χ*^2^/*df* = 1.32). We observed that spikelets from the GRFL and NPFL sites (Fig. 2, Supplementary Table S1) consistently produced fewer seeds relative to other sites. We ascribed this difference to overall reductions in habitat size, plant abundance, and panicle density. We were unable to quantify habitat size or *U. paniculata* abundance and panicle density during our collection missions. Nonetheless, the noticeable differences in panicle density (Fig. 1D) led us to develop a dichotomous qualitative predictor variable that identified locations as displaying either relatively low or high panicle density. We then included this variable in our models (Hilbe 2011). Subsequently, overdispersion was not apparent (Pearson *χ*^2^/*df* range = 0.97–0.99). We continued with analyses utilizing backward variable selection. We set *α* = 0.05 for variable retention, applied Akaike’s Information Criterion (AIC) to compare models (Burnham and Anderson 2002), and calculated exponentiated effect sizes (EES) for negative binomial models (Coxe 2022).

#### Seeds counts per spikelet

We counted the number of seeds produced within every spikelet then organized the data into an ordinally scaled response variable consisting of seven categories representing the number of spikelets containing 0, 1, 2, 3, 4, 5, or 6 seeds. We computed mean number of seeds per spikelet by calculating mean score response functions via weighted least squares estimation for categorical covariates (i.e. GPSZ, haplotype, relative panicle density, and dune type) and their interaction with the collection year. We then modelled the mean score response against categorical covariates using the weighted least square method and calculated effect size with Cohen’s *w* (Cohen 1988).

However, weighted least squares results in linearly dependent response functions for ordinally scaled response variables when evaluating continuous covariates (i.e. latitude, VFDI, FSDI, collection year) with large numbers of unique values. Maximum likelihood procedures are recommended instead (Stokes *et al.* 2012; SAS Institute 2019). Therefore, we implemented ordinal logistic regression with a cumulative logit link function. Preliminary analyses revealed that the assumption of proportional odds was invalid for FSDI, VFDI, and latitude but was satisfied for the year covariate. Subsequently, we fit a partial proportional odds cumulative logit model. We specified unequal slopes for the FSDI, VFDI, and latitude variables then generated effect sizes. This model also evaluated the potential effects of panicle density on seeds per spikelets by including the categorical density variable.

#### Counts of normal vs. abnormal seeds

We counted the number of normal and abnormal seeds within all spikelets then fit a generalized linear model with a binary logit link function. Normal seeds possess a well-formed embryo and endosperm with no major defects (e.g. cracks, pest damage). Abnormal seeds include one or more of the following: missing organs (i.e. embryo and/or endosperm), malformation of the seed and internal organs, or pest damage (Supplementary Fig. S2). We specified GPSZ and the phylo-geographic variable as classification variables. We then selected reference cell coding as the parameterization method and generated effect sizes. We compared models using AIC as described previously.

## Results

### Total normal seed count

**Figure 3.**
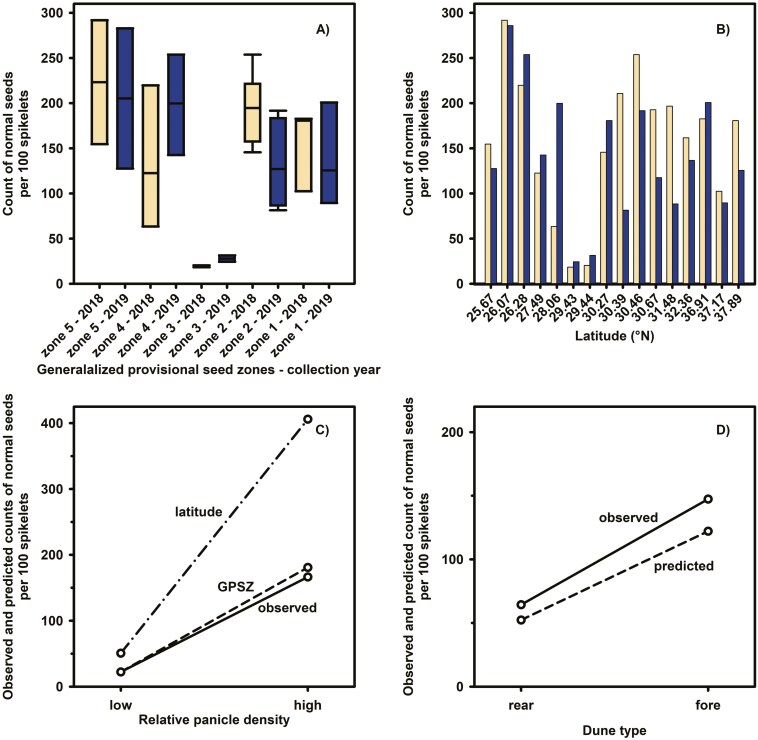
Count of normal seeds per 100 spikelets grouped by (A) Generalized Provisional Seed Zone and (B) latitude of occurrence for spikelets collected in 2018 (open bars) and 2019 (shaded bars). Generalized Provisional Seed Zones 1 and 5 represent the northernmost and southernmost zones, respectively. Effect plots of normal seed count per 100 spikelets for the (C) latitude and GPSZ models and (D) dune type model presented in Table 1. Ninety-five percent confidence intervals excluded from Fig. 2C and D for clarity.

Spikelets collected from GPSZ 1, 2, 4, and 5 produced on average about 125–225 seeds per 100 spikelets. However, plants in GPSZ 3 did not produce more than 32 normal seeds per 100 spikelets in either collection year. Seed production was higher in 2018 than 2019 for spikelets from GPSZ 1, 2, and 5, but the opposite was evident for spikelets from GPSZ 3 and 4. Seed production in GPSZ 3 and 4 was about 1.4 times greater in 2019 than 2018 (Fig. 3A).

**Table 1.** Negative binomial regressionr summary for models predicting count of total normal sea oats seeds per 100 spikelets.

Analysis	Variable	*Β*	SE	*EES* [95% CI]^a^	100 (*EES* − 1)^b^	Wald *χ*^2^	*df*	*P*	AIC
*GPSZ model* ^c^	density (0)	−2.02	0.21	0.13 [0.09, 0.20]	−87	96.24	1	<.0001	338.44
	FSDI	−0.35	0.19	0.71 [0.98, 1.17]	−29	3.52	1	.0607	
*Latitude model* ^d^	latitude	−0.03	0.02	0.97 [0.95, 1.00]	−3	3.03	1	.0820	337.59
	density (0)	−2.05	0.20	0.13 [0.09, 0.19]	−87	105.09	1	<.0001	
	FSDI	−0.34	0.18	0.71 [0.49, 0.99]	−29	3.68	1	.0552	

The density (0) covariate denotes a change from high to low density.

^a^Exponential effect size was calculated according to Coxe (2022).

^b^Percentage change of exponential effect size.

^c^Initial model excluded the latitude variable due to high correlation with the GPSZ variable.

^d^Initial model excluded the GPSZ variable due to high correlation with the latitude variable.

The GPSZ-based model provided considerable evidence (*P* < .0001) for a large deleterious effect of low relative plant density on seed count. For example, total normal seed counts from populations with low relative panicle density were 87% lower than seed counts from populations with high relative panicle density (Table 1). Likewise, for every 1-unit increase in FSDI seed counts decreased by about 29% for populations with low panicle density compared to those with high panicle density. However, the FSDI effect associated with this model warrants further exploration since *P* = .0607 (Table 1).

Seed production varied considerably across latitude, with spikelets collected at 26.07° N and 26.28° N consistently producing more than 200 seeds per 100 spikelets. Alternatively, total normal seed counts for spikelets from 29.43° N or 29.44° N were 1.3 to 15.4 times lower compared to spikelets from any other latitude (Fig. 3B). Inclusion of the panicle density covariate revealed that spikelets from sites with lower panicle density produced comparatively fewer seeds. The observed number of normal seeds produced in spikelets from low panicle density sites was roughly 7.0 times lower than spikelets from high panicle density locations (Fig. 3C).

The latitude-based model also provided evidence for a strong panicle density effect but less evidence for latitudinal or FSDI effects (Table 1). Regardless, all coefficients predicted decreases in seed counts. For example, total normal seed count was predicted to decrease by 3% for every 1-unit increase in latitude. A larger decrease was predicted for FSDI. Here, every 1-unit increase in FSDI was predicted to yield a 29% decrease in total normal seed count. Meanwhile, total normal seed count for spikelets from low panicle density sites was predicted to be 87% lower than seed counts for spikelets from high density areas (Table 1).

However, the change in AIC values (i.e. Δ_*i*_ = AIC_GPSZ_ − AIC_latitude_; 338.44 − 337.59 = 0.85; Table 1) between models was less than 2, thereby providing evidence that the simpler GPSZ model (Table 1) is the better approximating model. Similarly, effect plots (Fig. 3C) indicate a large overestimation of seed counts for the latitude model. The GPSZ model slightly overestimated total normal seed counts for populations with high relative panicle density.

The type of dune on which spikelets grow also influenced seed production. Total normal seed production was lower in spikelets from rear dune compared to foredune locations (Fig. 3D). However, there was no evidence for a dune type × collection year interaction (exponentiated effect size = 1.17, 95% CI = 0.39, 3.37; $\chi_{1}^{2}$ = 0.08; *P* = .7740). A subsequent main effects model provided evidence for a strong dune effect but not collection year effects (exponentiated effect size = 0.64, 95% CI = 0.37, 1.09; $\chi_{1}^{2}$ = 2.64; *P* = .1045). In this model, total normal seed production was predicted to increase by 2.3 times (95% CI = 1.34, 3.91; $\chi_{1}^{2}$ = 9.53; *P* = .0020) for spikelets from foredunes compared to rear dunes. However, this predicted effect may represent an underestimate (Fig. 3D).

### Seed counts per spikelet

#### Categorical covariates

**Figure 4.**
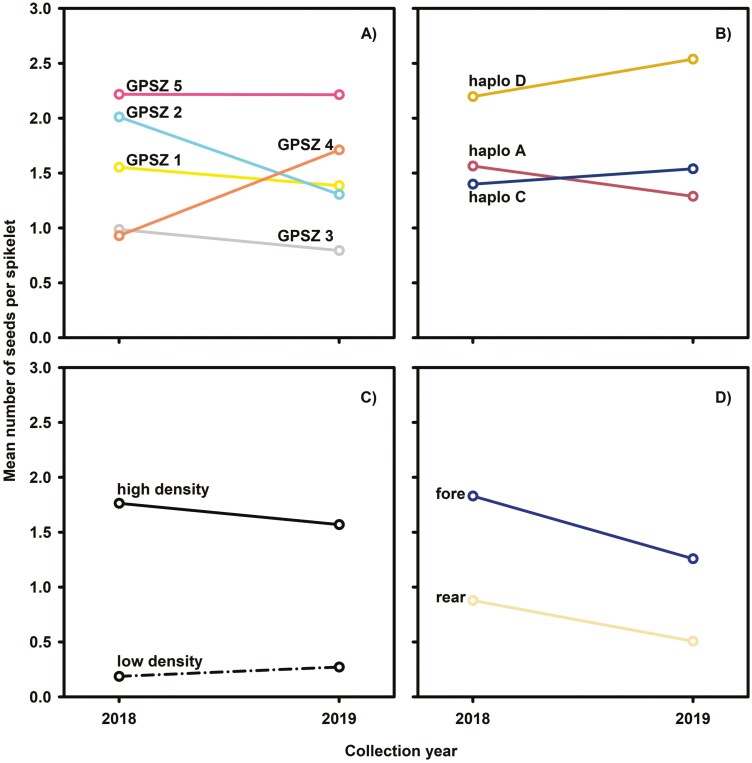
Effect plots for mean number of seeds per spikelet organized by (A) Generalized Provisional Seed Zone; (B) haplotype; (C) relative panicle density; and (D) dune type over two collection years. (A) Generalized Provisional Seed Zones 1, 2, 3, 4, and 5 denoted by yellow, blue, grey, orange, and pink lines, respectively. Generalized Provisional Seed Zones 1 and 5 represent the northernmost and southernmost zones, respectively. (B) Haplotypes A, C, and D denoted by red, blue, and gold lines, respectively. (C) High (solid line) and low (dash-dot line) relative panicle density. (D) Spikelets collected from plants growing on foredune (blue line) and rear dune (tan line).

The mean number of seeds per spikelet varied across GPSZ and ranged from 0.80 to 2.22. (Fig. 4A). However, some prominent interannual shifts in mean number of seeds per spikelet were evident. For example, within GPSZ 2, the mean number of seeds per spikelet decreased by about 1.5 times from 2018 to 2019. But the mean number of seeds per spikelet increased by roughly 1.8 times from 2018 to 2019 in GPSZ 4. Interestingly, the mean number of seeds per spikelet remained steady from year to year in GPSZ 5 (Fig. 4A). An interaction between each categorical covariate and collection year in terms of mean number of seeds per spikelet was evident (Fig. 4, Table 2). Ensuing single degree of freedom contrasts indicated negligible to small effects for about 62% of comparisons (Table 3). However, a medium GPSZ effect on mean number of seeds per spikelet was apparent when comparing zones 2 to 3–5 or zone 3 against 5 in 2018. For 2019, a few moderate to large GPSZ effects occurred between zones 1, 2, 3, and 5. Interannual contrasts detected medium collection year effects on mean number of seeds per spikelet in zones 2 and 4 (Table 3).

**Table 2.** Summary of models for sea oats seed counts per spikelet.

Predictor	Wald *χ*^2^	*df*	*P*
*Models for categorical predictor variables*			
GPSZ	404.68	4	<.0001
year	1.71	1	.1913
GPSZ × year	120.90	4	<.0001
haplotype	83.97	2	<.0001
year	0.81	1	.3691
haplotype × year	20.43	2	<.0001
panicle density	1698.56	1	<.0001
year	2.43	1	.1188
panicle density × year	16.02	1	<.0001
dune type	360.33	1	<.0001
year	107.41	1	<.0001
dune type × year	5.35	1	.0208
*Model for continuous variables and density*			
latitude	97.36	6	<.0001
VFDI	6.99	6	.3216
FSDI	68.71	6	<.0001
year	0.43	1	.51216
panicle density	405.97	1	<.0001

**Table 3.** Single degree of freedom orthogonal contrasts with associated effect sizes for categorical covariates examining seed counts per sea oats spikelets.

Contrast	Cohen’s *ω*^a^	Effect size^b^	*χ* ^2^	*P*	*n*
*GPSZ*					
1 vs. 2 in 2018	0.23	small	32.79	<.0001	600
1 vs. 3 in 2018	0.25	small	43.90	<.0001	700
1 vs. 4 in 2018	0.28	small	39.24	<.0001	500
1 vs. 5 in 2018	0.01	negligible	0.05	.8231	600
2 vs. 3 in 2018	0.47	medium	175.18	<.0001	800
2 vs. 4 in 2018	0.48	medium	136.17	<.0001	600
2 vs. 5 in 2018	0.35	medium	87.88	<.0001	700
3 vs. 4 in 2018	0.02	negligible	0.35	.5533	600
3 vs. 5 in 2018	0.38	medium	100.24	<.0001	700
4 vs. 5 in 2018	0.40	medium	80.74	<.0001	500
1 vs. 2 in 2019	0.12	small	10.76	.0010	700
1 vs. 3 in 2019	0.36	medium	92.33	<.0001	700
1 vs. 4 in 2019	0.09	negligible	3.87	.0492	500
1 vs. 5 in 2019	0.04	negligible	1.00	.3168	600
2 vs. 3 in 2019	0.52	large	213.69	<.0001	800
2 vs. 4 in 2018	0.20	small	23.60	<.0001	600
2 vs. 5 in 2019	0.16	small	17.75	<.0001	700
3 vs. 4 in 2019	0.24	small	35.99	<.0001	600
3 vs. 5 in 2019	0.63	large	275.02	<.0001	700
4 vs. 5 in 2019	0.17	small	13.69	.0002	500
2018 vs. 2019 in 1	0.07	negligible	3.32	.0685	600
2018 vs. 2019 in 2	0.35	medium	100.73	<.0001	800
2018 vs. 2019 in 3	0.09	negligible	6.12	.0134	800
2018 vs. 2019 in 4	0.33	medium	42.33	<.0001	400
2018 vs. 2019 in 5	0.05	negligible	1.71	.1913	600
*Haplotype*					
A vs. D in 2018	0.12	small	18.28	<.0001	1300
A vs. C in 2018	0.07	negligible	6.99	.0082	1500
D vs. C in 2018	0.24	small	23.16	<.0001	400
A vs. D in 2019	0.25	small	83.90	<.0001	1300
A vs. C in 2019	0.20	small	64.45	<.0001	1500
D vs. C in 2019	0.44	medium	79.04	<.0001	400
2018 vs. 2019 in A	0.11	small	29.46	<.0001	2400
2018 vs. 2019 in D	0.12	small	2.93	.0871	200
2018 vs. 2019 in C	0.04	negligible	0.81	.3691	600
*Relative panicle density*					
low vs. high density in 2018	0.86	large	1196.19	<.0001	1600
low vs. high density in 2019	1.03	large	1698.56	<.0001	1600
2018 vs. 2019 in low density	0.08	negligible	2.76	.0967	400
2018 vs. 2019 in high density	0.03	negligible	2.43	.1188	2800
*Dune type*					
foredune vs. rear dune in 2018	0.55	large	181.89	<.0001	600
foredune vs. rear dune in 2019	0.60	Large	360.33	<.0001	998
2018 vs. 2019 in foredune	0.29	Small	66.26	<.0001	799
2018 vs. 2019 in rear dune	0.37	Medium	107.41	<.0001	799

^a^Calculated as $\sqrt{\frac{\chi^{2}}{n}}$ according to Cohen (1988).

^b^ Cohen (1988) provides general guidelines for interpreting effect sizes when no discipline-specific criteria exist. The effect size guidelines for *ω* are as follows: negligible (< 0.10), small (0.10 < 0.30), medium (0.30 < 0.50), and large (> 0.50).

The mean number of seeds per spikelet ranged from 1.3 to 1.6 for haplotypes A and C while spikelets from haplotype D displayed a mean of 2.2 to 2.5 seeds per spikelet (Fig. 4B). On the contrary, remarkable shifts in the number of seeds per spikelet emerged when grouping spikelets by relative panicle density or dune type (Fig. 4C and D). For example, the mean number of seeds per spikelet was 5.4 to 8.9 times less in low panicle density sites than high density sites. Similarly, the mean number of seeds per spikelet was 1.4 to 2.4 times less in rear than foredune spikelets (Fig. 4C and D).

Nearly all comparisons of haplotypes within a collection year and collection years within a haplotype were negligible to small (Table 3). In contrast, the effects of panicle density and dune location on mean number of seeds per spikelets were large. Interannual differences in mean number of seeds per spikelets were negligible in the low and high panicle density sites. However, interannual differences in the fore and rear dune locations were larger (Table 3).

#### Continuous covariates

Most spikelets produced between zero to three seeds rather than four to six seeds across the latitudinal (Supplementary Fig. S3), drought (Supplementary Figs S4 and S5), and collection year (Supplementary Fig. S6A and B) gradients. However, 81% of spikelets collected from sites with low panicle density contained no seeds, while much fewer spikelets contained one (18%) or two (1%) seeds (Supplementary Fig. S6C). Spikelets collected from high panicle density sites displayed patterns for numbers of seeds like that of other continuous covariates (Supplementary Figs S3–S5, S6D). The ordinal regression model for continuous covariates provided compelling evidence for a positive effect of latitude, FSDI, and panicle density but not VFDI or collection year on the number of seeds per spikelets (Tables 2 and 4). For example, the odds of spikelets being in a category delineated by possessing fewer numbers of seeds compared to higher numbers of seeds increased by 1%–36% for each 1-unit increase in latitude (Table 4; percent change = (odds ratio − 1) × 100). Similarly, for each 1-unit increase in FSDI, the odds of spikelets possessing fewer numbers of seeds increased about 2.3–7.0 times. But the results for FSDI should be interpreted cautiously given the wide confidence intervals as the number of seeds per spikelets increased (Table 4). Panicle density produced a much larger effect on the number of seeds per spikelets than latitude or FSDI. The odds of spikelets collected from relatively low panicle density sites possessing fewer seeds compared to more seeds were nearly 18 times greater than the odds for spikelets from sites with relatively high panicle density (Table 4).

**Table 4.** Odds ratio estimates for statistically significant effects in the partial proportional odds cumulative logit model for continuous covariates, the categorical covariate panicle density, and the ordinal response variable of number of seeds per sea oats spikelets.

Effect	Odds ratio [95% CI]	Wald *χ*^2^	*P*
*Latitude*			
<1 seed per spikelet	1.04 [1.02, 1.06]	15.01	.0001
<2 seeds per spikelet	1.10 [1.07, 1.12]	60.75	<.0001
<3 seeds per spikelet	1.20 [1.15, 1.26]	70.94	<.0001
<4 seeds per spikelet	1.31 [1.18, 1.47]	23.82	<.0001
<5 seeds per spikelet	1.36 [1.01, 1.84]	4.05	.0442
*FSDI*			
<1 seed per spikelet	2.27 [1.78, 2.88]	44.49	<.0001
<2 seeds per spikelet	3.47 [2.51, 4.79]	57.13	<.0001
<3 seeds per spikelet	3.53 [2.00, 6.37]	17.49	<.0001
<4 seeds per spikelet	3.20 [0.75, 13.56]	2.49	.1146
<5 seeds per spikelet	6.98 [0.08, 619.15]	0.72	.3958
*Panicle density*			
Low vs. high	17.77 [13.43, 23.50]	405.97	<.0001

The highest number of seeds per spikelets (i.e. 6) was used as the reference category when dichotomizing the response variables.

### Counts of normal vs. abnormal seeds

The frequency of normal seeds was 2.5 to 22.8 times (median = 8.9) greater than that of abnormal seeds across all covariates. Greater variation in relative frequency of normal to abnormal seeds was observed for the VFDI and FSDI covariates compared to the remaining covariates (Supplementary Fig. S7). However, only FSDI displayed a significant effect. The odds of normal seeds decreased by 43% for every 1-unit increase in FSDI (Table 6). Notably, the range in frequency of normal (86%–90%) and abnormal (10%–14%) seeds was similar between spikelets collected from low and high panicle density locations (Supplementary Fig. S7D). Akaike’s Information Criterion indicated that the GPSZ-based rather than the latitude-based model was the better approximating model when assessing counts of normal versus abnormal seeds (Table 5). Ensuing orthogonal contrasts revealed that the odds of normal seeds are about 1.9–2.7 times greater than abnormal seeds for spikelets from GPSZ 2 compared to other GPSZ. Interestingly, spikelets from GPSZ 3, which encompasses the low panicle density sites used in this study, produced slightly fewer normal seeds and more abnormal seeds compared to the other GPSZ. However, odds ratios indicated that such differences were not statistically significant (Table 6).

**Table 5.** Logistic regression summaries of counts of normal and abnormal seeds per 100 sea oats spikelets for models containing either the GPSZ or latitude as predictors.

Analysis	Predictor	Wald *χ*^2^	*df*	*P*	AIC
*GPSZ model* ^a^	GPSZ	37.23	4	<.0001	3449.23
	haplotype	2.92	2	.2321	
	year	0.04	1	.8479	
	VFDI	3.60	1	.0578	
	FSDI	8.11	1	.0044	
*Latitude model* ^b^	latitude	0.31	1	.5759	3484.81
	haplotype	6.29	2	.0430	
	year	1.05	1	.3067	
	panicle density	0.45	1	.5014	
	VFDI	6.06	1	.0139	
	FSDI	0.62	1	.4300	
*Dune-type model*	dune	4.00	1	.0456	
	year	0.38	1	.5400	
	dune × year	3.99	1	.0457	

^a^Model excluded the latitude variable due to high correlation with the GPSZ variable. Note that GPSZ 3 encompasses the populations with low panicle density. Therefore, the model sets the density parameter to 0 since this variable forms a linear combination with GPSZ 3.

^b^Model excluded the GPSZ variable due to high correlation with the latitude variable.

**Table 6.** Odds ratio estimates for orthogonal contrasts for statistically significant predictors from logistic regression analyses comparing normal to abnormal sea oats seeds.

Contrast	Odds ratio [95% CI]
*GPSZ*	
2 vs. 1	2.31 [1.65, 3.24]
3 vs. 1	0.87 [0.48, 1.51]
4 vs. 1	1.23 [0.85, 1.78]
5 vs. 1	0.86 [0.65, 1.14]
2 vs. 3	2.70 [1.45, 5.03]
2 vs. 4	1.88 [1.31, 2.68]
2 vs. 5	2.70 [1.93, 3.73]
3 vs. 4	0.69 [0.37, 1.31]
3 vs. 5	0.93 [0.55, 1.78]
4 vs. 5	1.43 [1.00, 2.10]
*FSDI*	0.57 [0.38, 0.84]

Sufficient statistical evidence also existed supporting a dune × year interaction for the dune type model when assessing normal versus abnormal seeds (Table 5). The odds of normal seeds compared to abnormal seeds were nearly 2.5 (95% CI [1.52, 4.06]) times greater in foredunes than rear dunes during the 2018 spikelet harvest. The odds dropped to 1.3 (95% CI [0.88, 1.94]) in 2019 and were not statistically different from one. The odds of normal compared to abnormal seeds in 2018 were 13% (95% CI [0.54, 1.38]) lower than that of spikelets collected in 2019 from rear dune locations and were not statistically significant. But in spikelets collected from foredune locations, the odds of a normal compared to abnormal seed in 2018 were 55% (95% CI [0.30, 0.69]) lower than that of spikelets harvested in 2019.

## Discussion

The number of normal seeds produced by a species represents a key component driving regeneration, fitness, and ultimate population dynamics. Yet plants are confronted with numerous biotic and abiotic factors that filter the number of seeds produced *in planta* and that will ultimately be realized as seedlings (Fenner and Thompson 2005; Baskin and Baskin 2022). In this study we investigated covariates that may influence seed production of an important coastal dune grass with a large geographic distribution. We found that seed production varied considerably across the *U. paniculata* range. Relative panicle density and dune system from which spikelets were collected displayed the largest effects of any covariates on total normal seed production and number of seeds per spikelet but not on the proportion of normal to abnormal seeds. However, environmental factors such as drought during the seed developmental period should not be discounted when considering the influence of abiotic factors on seed production.

### Panicle Density as a Driver of Seed Production

Relative panicle density accounted for the largest effects on total normal seed production and seeds per spikelet across the *U. paniculata* distribution. Our study included two proxy covariates for the measurement of panicle density. In the first, two sites located at 29.43° N and 29.44° N latitude, which also represented GPSZ 3, displayed fewer panicles relative to other sites (Fig. 1D). Both sites are considered critically eroded beaches that have experienced heavy and continued sand loss (Olsen Associates 2022; Clark and Weeks 2023). A *U. paniculata-*dominated foredune is absent at these sites. Instead, the sloped beach intersects with an eroded vertical dune cliff. The narrow dune crest on this cliff is dominated by forbs, low-growing woody shrubs, and rhizomatous palms with *U. paniculata* intermixed sporadically (Tia Tyler, pers. obs.), suggesting that the existing dune represents a former rear dune (Johnson and Barbour 1990; Knight *et al.* 2011). The second proxy consisted of intact rear dune locations where the density of *U. paniculata* decreases compared to adjacent foredunes (Fig. 1D; Wagner 1964; Johnson and Barbour 1990; Knight *et al.* 2011).

*Uniola paniculata* is thought to be an outcrossing and wind-pollinated species (Franks *et al.* 2004). This inference makes sense when connecting relative panicle density to seed production and the number of seeds per spikelet. Additionally, *U*. *paniculata* flowers collected from dunes in North Carolina produced abundant pollen and exhibited high stigmatic pollen loads (Wagner 1964). Therefore, pollen capture may not necessarily be a limiting factor. But the situation changes when the density of *U. paniculata* individuals decreases. Fewer individuals result in fewer panicles, fewer spikelets, and fewer androecia that can produce pollen. Importantly, reduced density also results in fewer gynoecia that can receive pollen, be successfully fertilized, and produce normal seeds. A decrease in density is compounded by short pollen transport distances. For example, Friedman and Barrett (2009) contend that most grass pollen is deposited near donor plants due to a leptokurtic distribution centred on pollen source plants. A consequence of this is that seed set is predicted to decrease rapidly as the distance between pollen donors and recipients increases. We view this as an appropriate explanation for the large decrease in seed production and number of seeds per spikelet in *U. paniculata* populations where panicle density is low. This perspective is consistent with work by Hester and Mendelssohn (1987) showing that seed production was severely reduced in locations with sparse *U. paniculata* cover. Colosi (1980) also described low to no seed production for populations occurring at about 29° N. This agrees with our finding of large decreases in seed production for reduced populations at the same latitude.

### Influence of eco-spatial covariates on seed production

Seed production is an important life-history trait that is expected to vary across eco-spatial zones or latitude given interannual micro-climatic differences, habitat-related stressors, and human-induced habitat changes across continental-scale gradients (Fenner and Thompson 2005; De Frenne *et al.* 2013; Baskin and Baskin 2022). For example, several authors report that populations growing in eco-spatial zones displaying warmer, wetter conditions or generally less stressful conditions displayed greater normal seed production than populations growing in cooler, drier zones or zones experiencing higher degrees of stress (García *et al.* 2000; Miller and Cummins 2001; Ayari *et al.* 2011). Additionally, Ayari *et al.* (2011), Jump and Woodward (2003), and García *et al.* (2000) found linear and quadratic relationships between normal seed production and latitude. These authors attribute such relationships to harsher conditions at the southern end of the tested gradient (Ayari *et al.* 2011) or at the peripheries rather than the distributional core of the test species (García *et al.* 2000; Jump and Woodward 2003).

However, in the current study, insufficient statistical evidence existed to support an eco-spatial zone (i.e. GPSZ) effect on the count of normal *U*. *paniculata* seeds. Likewise, counts of normal seeds were predicted to decrease by a small amount (~ 3%) as latitude increased. But the latitude effect only produced marginal statistical support, and the latitudinal-based model was not a good predictor of normal seed counts (Table 1, Fig. 3C). Similarly, there was no latitudinal trend in seed production for a wide-ranging species of *Cirsium* (Jump and Woodward 2003). Our results contradict findings indicating that northern *U. paniculata* populations tend to produce more normal seeds than southern populations (Hester and Mendelssohn 1987 and references therein). Nonetheless, such a small decrease in normal seed production across a wide distributional gradient, as presented in our study, may not necessarily be detrimental since locations with high panicle density have a tendency to produce copious viable seeds that germinate and produce normal seedlings after experiencing various types of stress and environmental conditions (Wagner 1964; Westra and Loomis 1966; Seneca 1972; Burgess *et al.* 2002; Pérez and Kane 2017; Egesa *et al.* 2023).

We suspect that the lack of a detected eco-spatial effect and the small latitudinal effect on normal seed production may be related to the fact that *U. paniculata* populations are continuously confronted with a combination of stressful conditions, like high temperatures, low soil moisture, and high salinity, across its narrowly linear yet wide geographic distribution (Johnson and Barbour 1990; Knight *et al.* 2011). In contrast, populations of other tested species may experience better or worse conditions at one end or at the extremes rather than the centre of a latitudinal gradient. It is likely that current levels of abiotic stressors experienced by *U*. *paniculata* may not be sufficient to substantially depress normal seed production in a species that has evolved under persistently challenging conditions of coastal dunes. This perspective is different from the abundant center model of biogeography that predicts performance will be better towards the centre of a species range owing to more suitable conditions there than at the range peripheries (Brown 1984; Sagarin and Gaines 2002; Daco *et al.* 2021).

Interestingly, the southernmost populations, delineated by GPSZ 5 and haplotype D, tended to produce a higher mean number of seeds per spikelet than populations in other zones or haplotypes. Combining this with data showing that populations at the southernmost latitudes (Fig. 3B) produced on average more normal seeds than populations at other latitudes suggests that the southern peninsula of Florida may be an area of contrastingly high seed production. This conforms with the hypothesis of a genetic diversity hotspot for *U. paniculata* in the southern peninsula of Florida that acted as a refugia during the last glacial maximum (Hodel and Gonzalez 2013). However, our results do not entirely parallel a previous *U. paniculata* seed production study. Hester and Mendelssohn (1987), citing regression analysis by Colosi (1980), state that seed production decreased moving southward from Virginia (mean = 1.5 seeds per spikelet) to southern Florida (mean = 0.6 seeds per spikelet), but some Gulf coast populations (ca. 26–27° N) displayed mean seed per spikelet values between 1 and 2. The variation in mean seed numbers per spikelet between studies could be the result of robust interannual plasticity (Bazzaz *et al.* 2000; Fenner and Thompson 2005).

The proportion of abnormal seeds across all covariates (range 4% to 28%, mean = 10.9%, 95% CI = 9.6, 12.3) was not unusual for wild species (Kärkkäinen 1999; Pérez 2014). Moreover, we did not observe drastic differences in the relative frequency between normal and abnormal seeds when data were grouped by panicle density. Likewise, the odds of normal seeds to abnormal seeds in GPSZ 3, which encompassed the sites with low panicle density, were not statistically different than the odds for any other zone. We speculate that the degree of abnormal seed production found across the continental distribution of *U. paniculata* is not necessarily related to potentially low genetic diversity or inbreeding depression. Indeed, Hodel and Gonzalez (2013) report a high degree of genetic diversity among *U. paniculata* populations across its North American distribution. Perhaps it is more important to focus on environmental factors, such as increases in drought conditions during the flowering to seed production pheno-phase, along with associated interannual variation while developing explanations for changes in the proportion of normal to abnormal seeds. Nonetheless, practitioners working with unconditioned seed lots may want to consider incorporating an abnormal seed compensation factor of 10% to 30% when calculating seeding rates for propagation of *U*. *paniculata*.

## Conclusions

Species exposed to continuously harsh conditions across their continental-scale distribution may be less susceptible to decreases in seed production compared to species that experience stressful conditions in certain parts of their range. Nonetheless, more severe types of stress, like extreme drought or heat waves during reproductive pheno-phases, should not be ignored. Apparently, non-climatic factors, such as population density, may be more persistent drivers of reduced seed production especially in fragmented populations that produce seeds during the same time frame across their range. Likewise, substantial interannual variation in seed production should be expected across a large spatial scale, and prospective studies should take such variation into consideration to obtain a better sense of how seed production fluctuates. Consequently, relationships between population density, interannual variation, higher degrees of stress, and seed production deserve further attention. Finally, for *U*. *paniculata*, we hypothesize that a threshold panicle density exists below which seed production decreases substantially. This information could enhance decision-making for seed-based coastal dune restoration and stabilization programs across a large portion of the US Atlantic and Gulf coasts. Therefore, clarifying such a threshold will be the goal of future studies. We conclude that practitioners involved in dune restoration activities should assess relative panicle density of sea oats at donor sites when creating seed collection plans and may consider sites with low panicle density as priority augmentation targets.

## Supplementary Material

plaf021_suppl_Supplementary_Tables_S1_Figures_S1-S7

## Data Availability

The data underlying this article are available in the Zenodo Digital Repository, at https://doi.org/10.5281/zenodo.13386933
