# Supplementary material for: Seeing right through it: X-ray analyses of Uniola paniculata L. spikelets reveal seed production patterns across a wide spatial distribution
Source: AoB Plants. 2025 Apr 12;17(4):plaf021. doi: 10.1093/aobpla/plaf021 (PMC12285731; doi:10.1093/aobpla/plaf021)
Supplement: plaf021_suppl_Supplementary_Tables_S1_Figures_S1-S7 [file plaf021_suppl_supplementary_tables_s1_figures_s1-s7.pdf]

Supporting Information

Table S1. List of *Uniola paniculata* L. (Sea oats) collections locations and continuous covariates used to assess variation in seed production along a continental-scale gradient.

Site Code	Collection Location	Permit Agency[z]	Latitude (°N)	VFDI[y]		FSDI[x]		Fore Dune Collection		Rear Dune Collection	
				2018	2019	2018	2019	2018	2019	2018	2019
CIVA	Chincoteague Nat. Wildlife Refuge (Accomack County, VA)	USFWS	37.89	1.00	0.00	0.14	0.09	Yes	Yes	No	No
KIVA	Kiptopeke State Park (Cape Charles, VA)	VDCR	37.17	1.00	0.00	0.14	0.00	Yes	Yes	No	No
FLVA	First Landing State Park (Virginia Beach, VA)	VDCR	36.91	1.00	0.00	0.14	0.00	Yes	Yes	No	No
FFNC	Fort Fisher State Recreation Area (Kure Beach, NC)	NCDCM	-[w]	-	-	-	-	No	Yes	No	Yes
HISC	Hunting Island State Park (Hunting Island, SC)	SCHEC	32.36	2.22	1.56	0.36	0.77	Yes	Yes	Yes	No
SIGA	Sapelo Is. Nat. Estuarine Research Reserve (Townsend, GA)	SINERR	31.48	2.33	0.78	0.14	0.77	Yes	Yes	Yes	Yes
FCFL	Fort Clinch State Park (Fernandina Beach, FL)	FLDEP	30.67	1.33	0.00	0.00	1.15	Yes	Yes	Yes	Yes
LTFL	Little Talbot Island State Park (Jacksonville, FL)	FLDEP	30.46	0.78	0.00	0.00	0.95	Yes	Yes	No	Yes
HBFL	Henderson Beach State Park (Destin, FL)	FLDEP	30.39	0.00	0.56	0.00	0.71	Yes	Yes	No	Yes
CHFL	Camp Helen State Park (Panama City Beach, FL)	FLDEP	30.27	0.00	0.44	0.00	0.71	Yes	Yes	No	No
NPFL	North Peninsula State Park (Ormond by the Sea, FL)	FLDEP	29.44	0.11	0.00	0.00	0.00	Yes	Yes	No	No

GRFL	Gamble Rogers Memorial State Park (Flagler Beach, FL)	FLDEP	29.43	0.11	0.00	0.00	0.00	Yes	Yes	No	No
HIFL	Honeymoon Island State Park (Dunedin, FL)	FLDEP	28.06	0.58	0.00	0.15	0.00	Yes	Yes	No	No
FPFL	Fort Pierce Inlet State Park (Fort Pierce, FL)	FLDEP	27.49	0.42	0.92	0.10	0.00	Yes	Yes	No	No
DWFL	Delnor-Wiggins Pass State Park (Naples, FL)	FLDEP	26.28	1.33	0.00	0.35	0.00	Yes	Yes	No	No
MJFL	Dr. Von D. Mizell-Eula Johnson State Park (Dania Beach, FL)	FLDEP	26.07	0.92	0.50	0.10	0.00	Yes	Yes	No	No
BBFL	Bill Baggs Cape Florida State Park (Key Biscayne, FL)	FLDEP	25.67	1.67	0.75	0.35	0.00	Yes	Yes	No	No

[z]Permits obtained prior to spikelet collection from the following agencies: FLDEP = Florida Department of Environmental Protection; SINERR = Sapelo Island National Estuarine Research Reserve; SCHEC = South Carolina Department of Health and Environmental Control; NCDCM = North Carolina Division of Coastal Management; VDCR = Virginia Department of Conservation and Recreation; USFWS = United States Fish and Wildlife Service.

[y]VFDI = drought index calculated during the *U. paniculata* vegetative through floral initiation phases.

[x]FSDI = drought index calculated during the *U. paniculata* post-floral initiation through seed maturation phases.

[w]This population was excluded from total normal and normal vs. abnormal seed counts given one year of spikelet collection. But data from this population was included in dune location analyses.

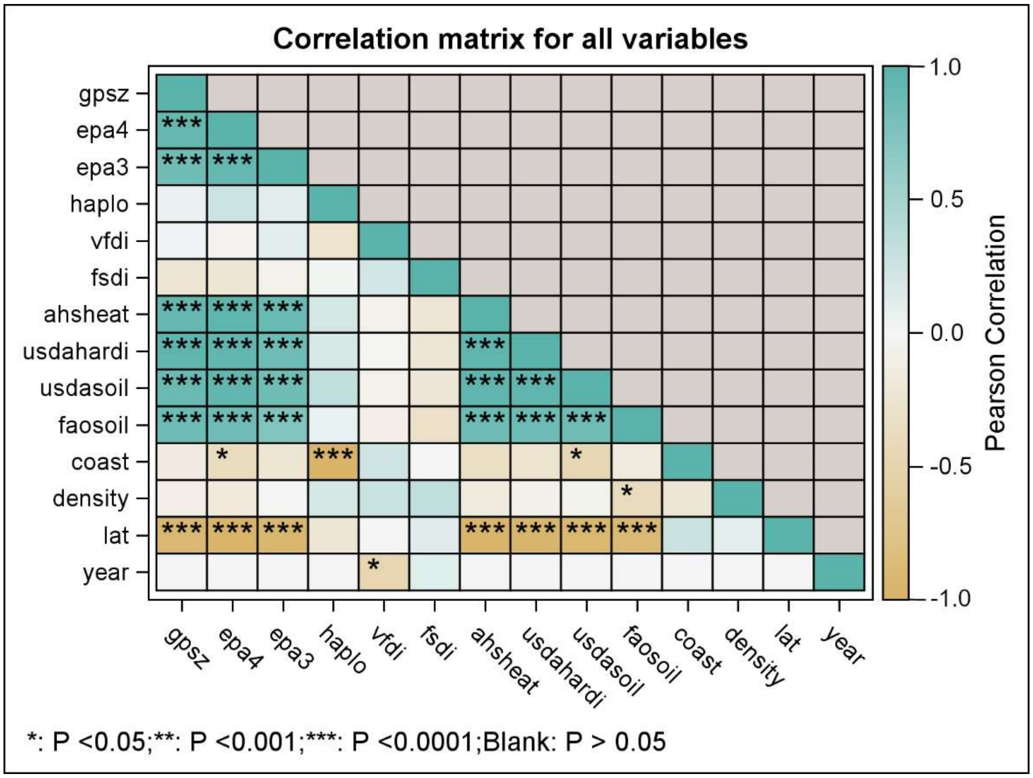

Figure S1. Pearson's correlation coefficients between initial covariates for assessing variation in *Uniola paniculata* (Sea oats) seed production. The variables are: GPSZ = general provisional seed zone (Bower *et al.* 2014); epa4 = U.S. Environmental Protection Agency level IV ecoregion (USEPA 2023); epa3 = U.S. Environmental Protection Agency level III ecoregion (USEPA 2023); haplo = phylo-geographic grouping of populations based on cpDNA variation (Hodel and Gonzalez 2013); vfdi = a drought index calculated during the *U. paniculata* vegetative through floral initiation phases (NDMC 2024; Pérez 2014); fsdi = a drought index calculated during the *U. paniculata* floral initiation through seed maturation phases (NDMC 2024; Pérez 2014); ahsheat = American Horticultural Society heat zones (AHS 1997); usdahardi = U.S. Department of Agriculture plant hardiness zones; usdasoil = U.S. Department of Agriculture soil classifications (USDA 2012); faosoil = Food and Agriculture Organization of the United Nations soil classifications (FAO-UNESCO 1975); coast = Gulf or Atlantic coast populations; density = qualitative factor delimiting populations with relatively low or high panicle density; latitude = angular distance north of the equator; year = collection year.

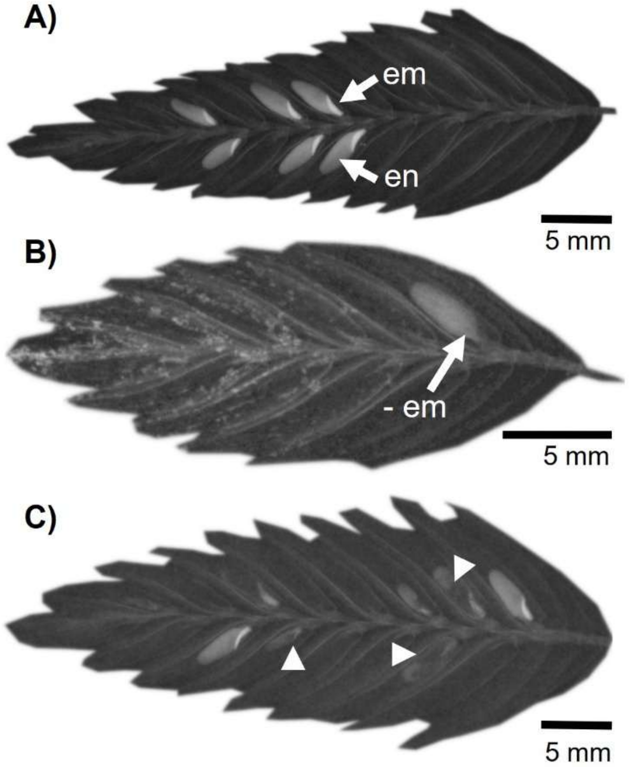

Figure S2. Images of normal (A) and abnormal (B) *Uniola paniculata* caryopses referred to as seeds within spikelets. em = embryo, en = endosperm, - em = seed with missing embryo. Triangles in (C) denote other defective seeds showing signs of organ and tissue abortion. Two normal and five aborted seeds can be seen in (C).

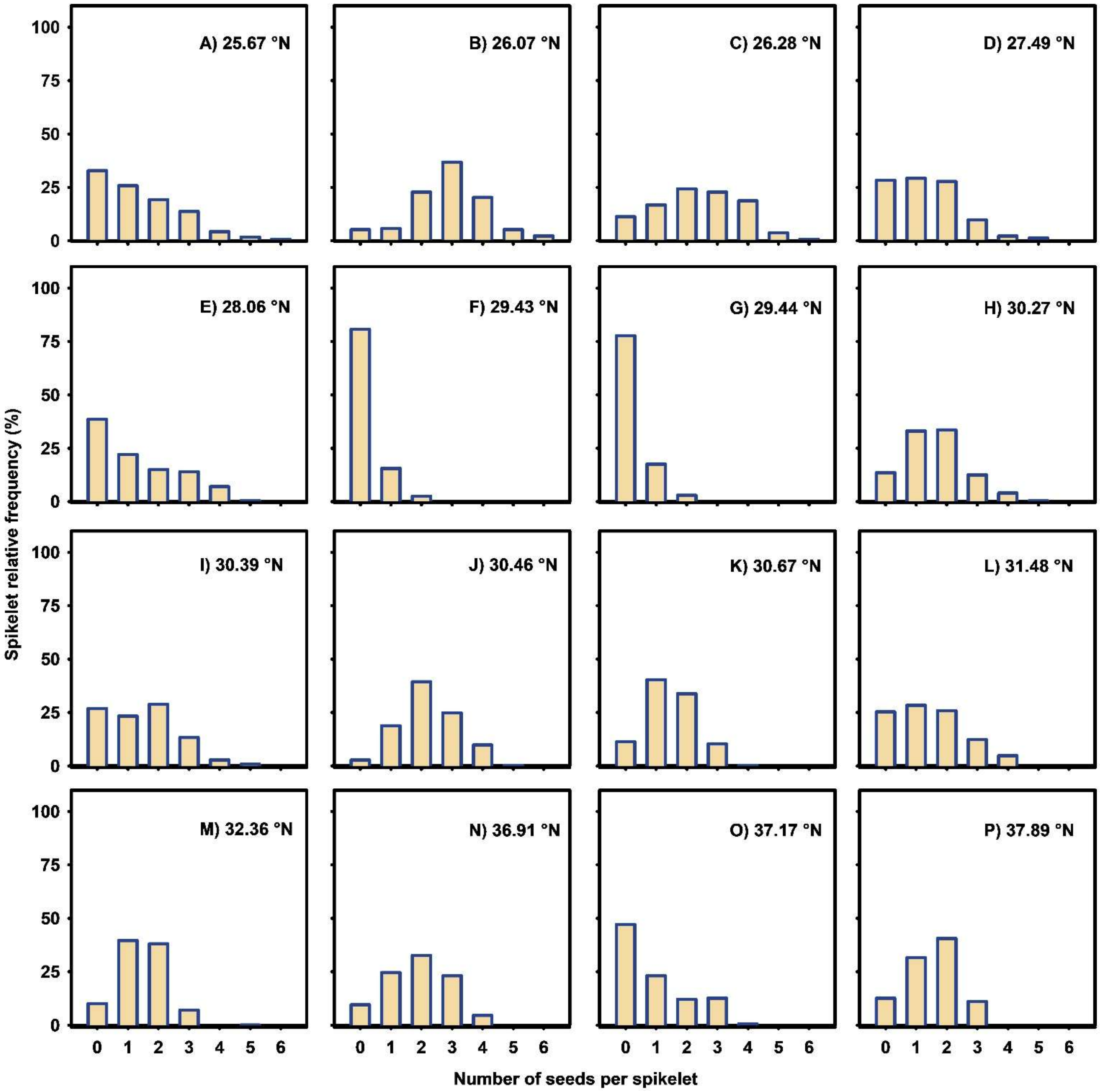

Figure S3. The relative frequency of sea oats spikelets containing 0, 1, 2, 3, 4, 5, or 6 seeds across a wide latitudinal gradient with n = 200 spikelets at each latitude.

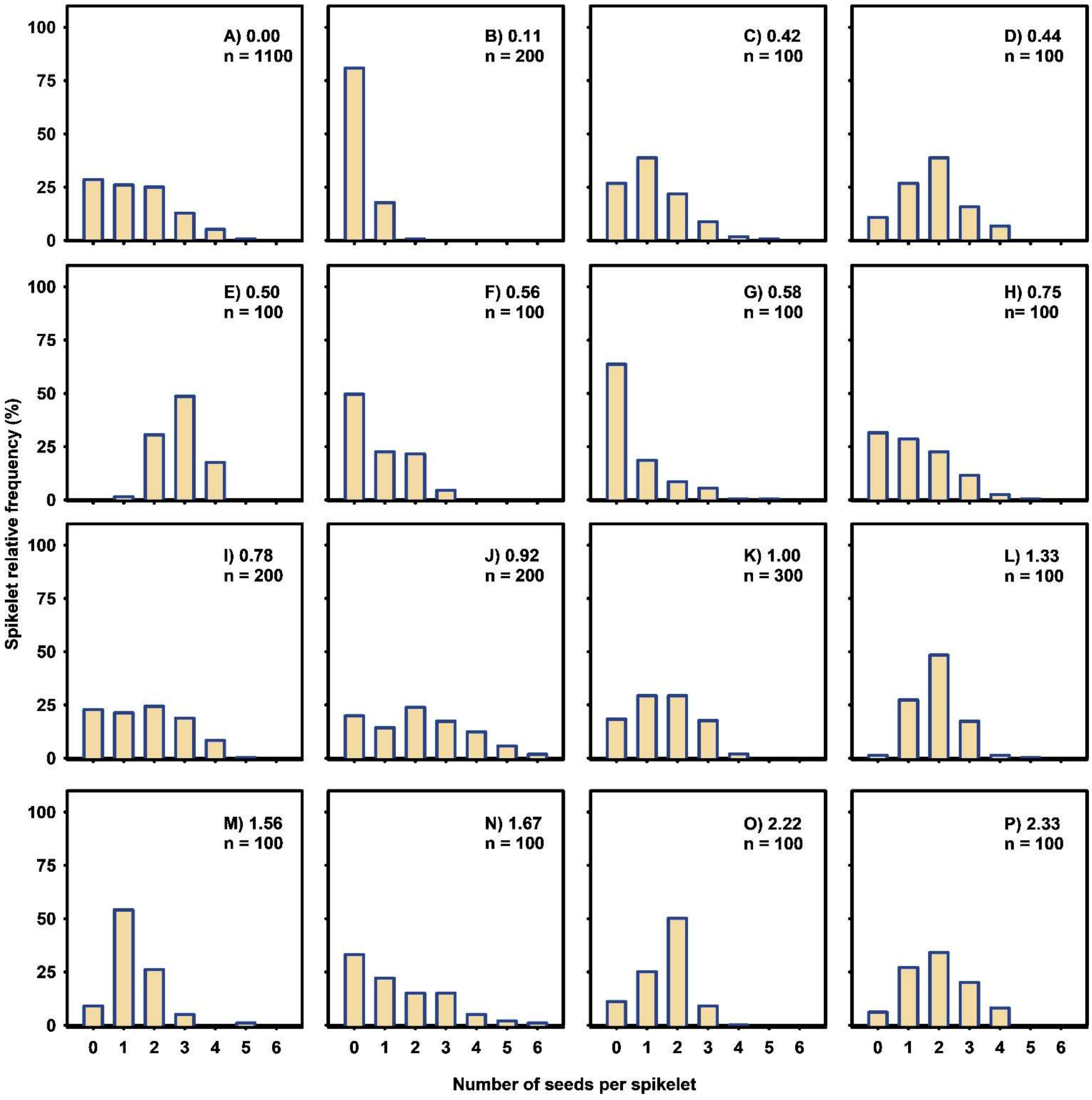

Figure S4. The relative frequency of sea oats spikelets containing 0, 1, 2, 3, 4, 5, or 6 seeds across a five-point drought index (abnormally dry conditions = 1, moderate drought = 2, severe drought = 3, extreme drought = 4, or exceptional drought =5) calculated during the vegetative to floral initiation pheno-phases (VFDI). Numbers in the upper right portion of each graph show the calculated drought index value.

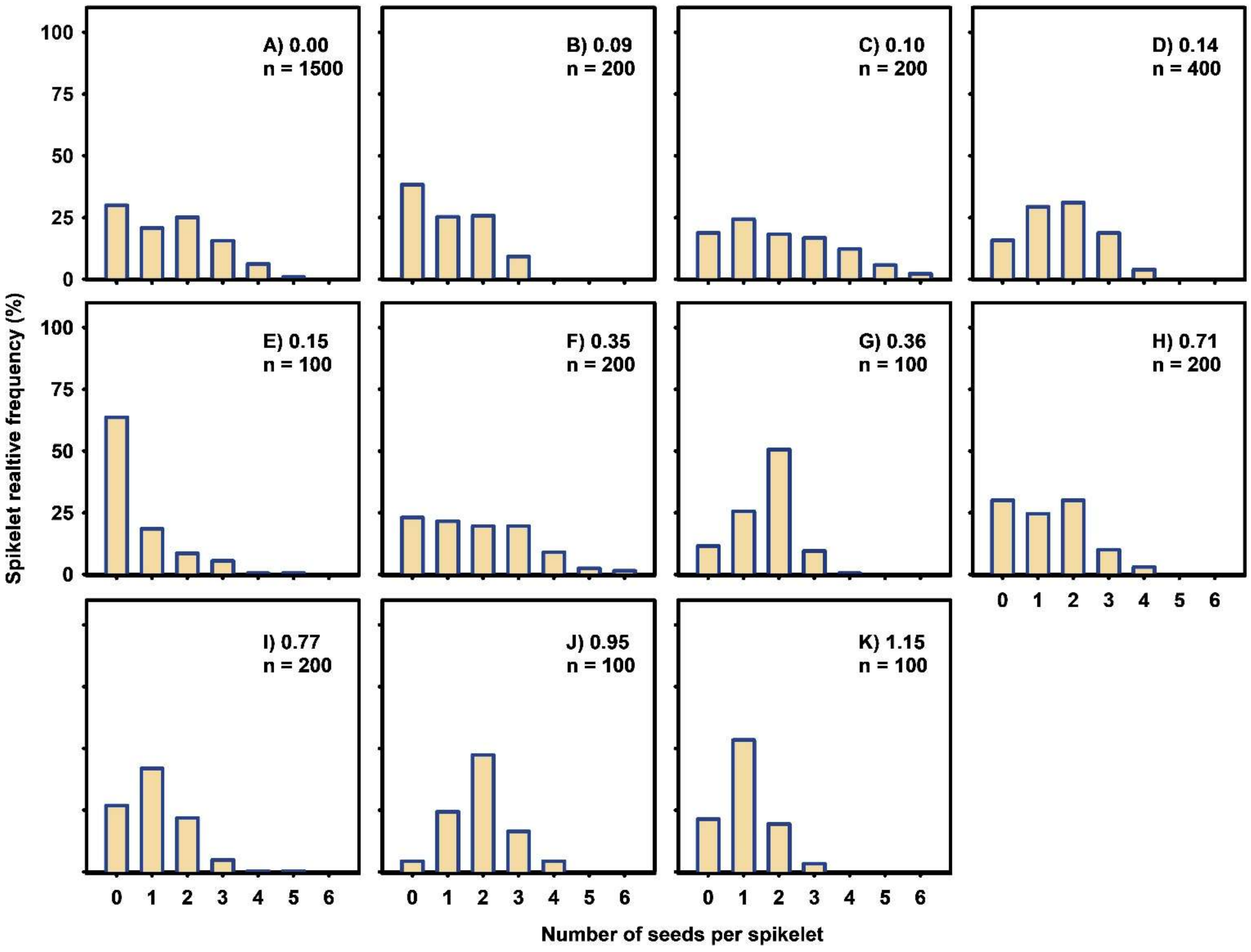

Figure S5. The relative frequency of sea oats spikelets containing 0, 1, 2, 3, 4, 5, or 6 seeds across a five-point drought index (abnormally dry conditions = 1, moderate drought = 2, severe drought = 3, extreme drought = 4, or exceptional drought =5) calculated during the post-floral initiation through seed maturation pheno-phases (FSDI). Numbers in the upper right portion of each graph show the calculated drought index value.

A bar graph of the number of spikelets containing from zero to six seeds across different drought levels experienced by sea oats plants during the vegetative to floral initiation phases.

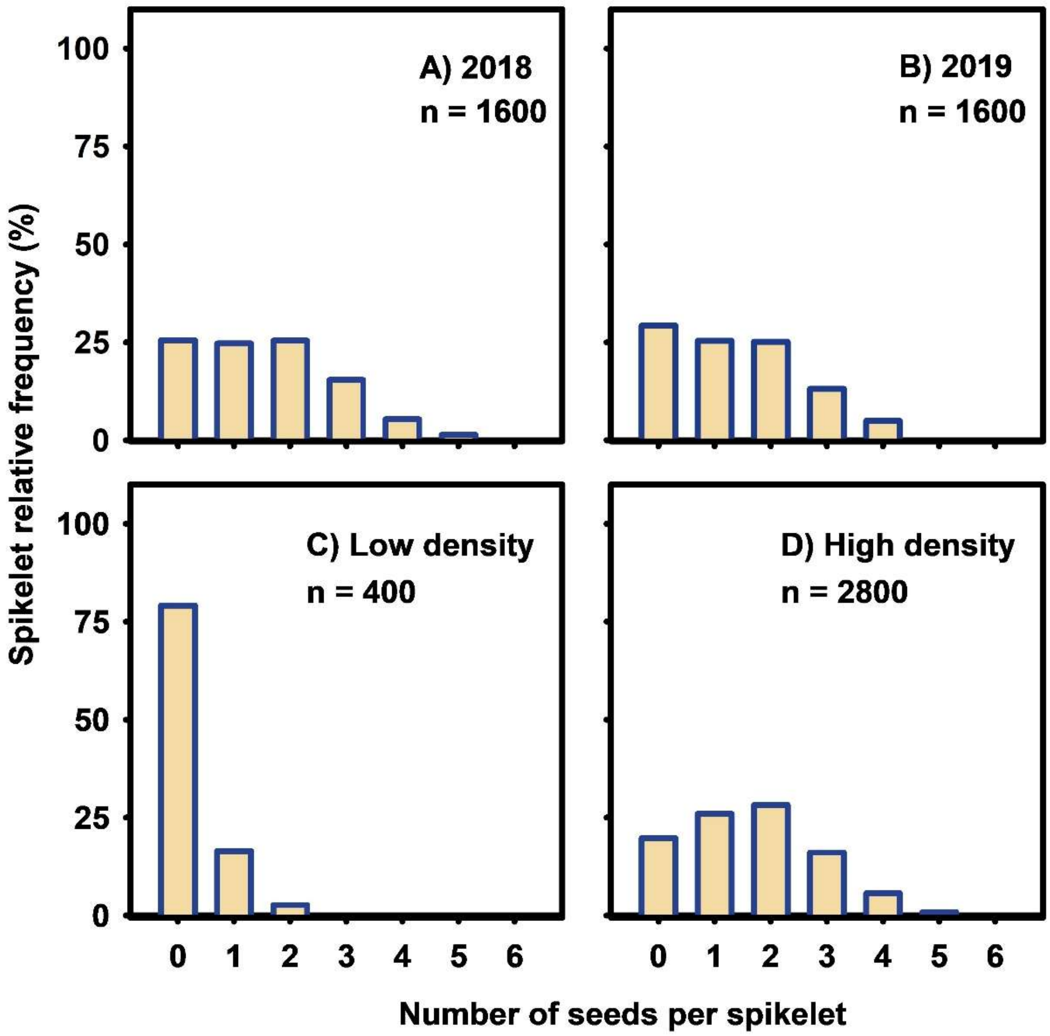

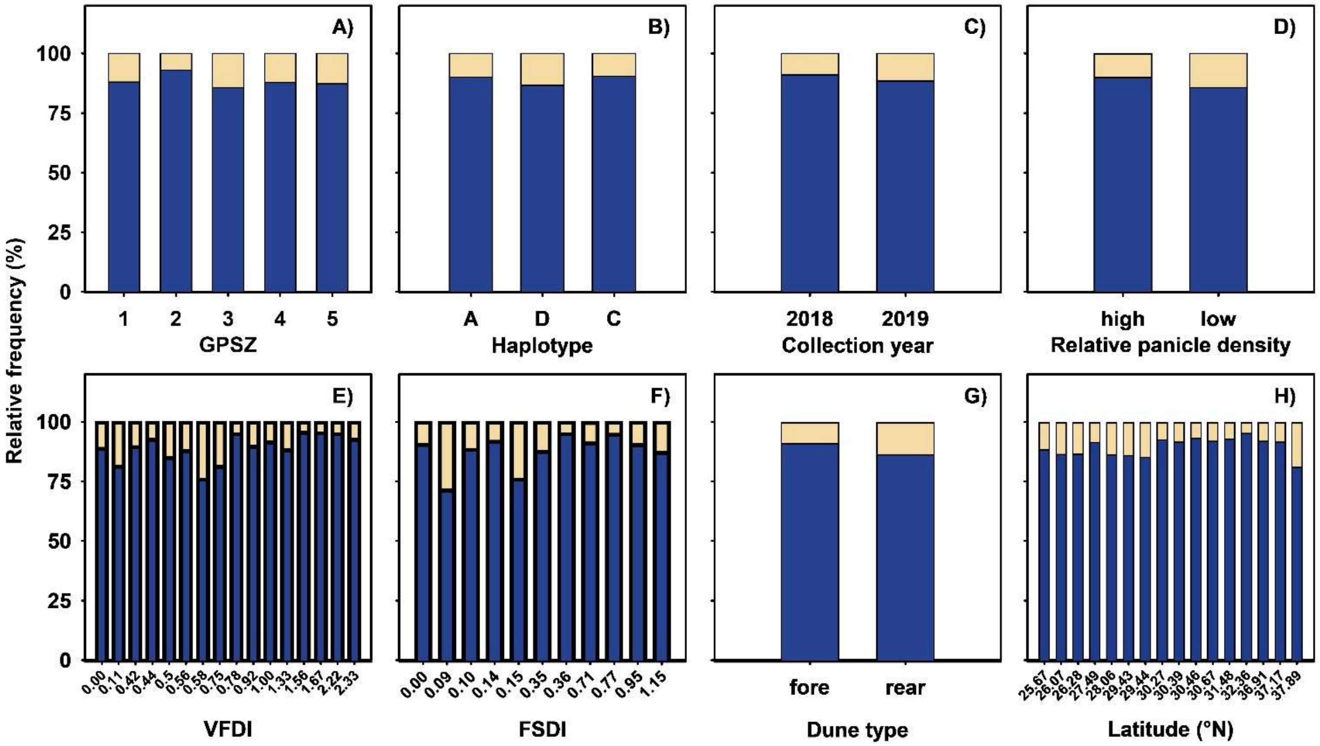

Figure S7. Relative frequency of abnormal (tan bar) and normal (blue bar) seeds per 100 spikelets for spikelets (n = 5,344) grouped by Generalized Provisional Seed Zone (A), haplotype (B), collection year (C), relative panicle density (D), vegetative to flowering drought index, VFDI (E), flowering to seed production drought index, FSDI (F), and dune type (G). Sample sizes for covariates in A-D, F, G = 5,344 and E = 1,901.

References

AHS, The American Horticultural Society. 1997. Heat zone map developed. Available at https://ahsgardening.org/about-us/news-press/cool_timeline/heat-zone-map-developed/ (accessed March 10 2010).

Bower A, St. Clair J, Erickson V. 2014. Generalized provisional seed zones for native plants. *Ecological Applications* 24: 913-919.

FAO-UNESCO. 1975. FAO-UNESCO soil map of the world 1:5,000,000 Volume II North America. Available at https://www.fao.org/soils-portal/data-hub/soil-maps-and-databases/faounesco-soil-map-of-the-world/en/ (accessed Oct 16 2019).

Hodel RG, Gonzalez E. 2013. Phylogeography of sea oats (*Uniola paniculata*), a dune-building coastal grass in southeastern North America. *Journal of Heredity* 104: 656-665.

NDMC, National Drought Mitigation Center. 2024. U.S. Drought Monitor. Available at https://droughtmonitor.unl.edu/Maps/MapArchive.aspx (accessed Dec. 1 2019).

Pérez H. 2014. Do habitat and geographic distribution influence decreased seed viability in remnant populations of a keystone bunchgrass? *Ecological Restoration* **32,** 295-305.

USDA, United States Department of Agriculture. 2012. USDA Plant Hardiness Zone Map, 2012. Available at https://planthardiness.ars.usda.gov/ (accessed Dec 10 2019).

USEPA, United States Environmental Protection Agency. 2023. Level III and IV ecoregions of the continental United States. Available at https://www.epa.gov/eco-research/level-iii-and-iv-ecoregions-continental-united-states (accessed Dec. 1 2019).
